# Tunable Multiple Plasmon-Induced Transparencies Based on Asymmetrical Graphene Nanoribbon Structures

**DOI:** 10.3390/ma10070699

**Published:** 2017-06-26

**Authors:** Chunyu Lu, Jicheng Wang, Shubin Yan, Zheng-Da Hu, Gaige Zheng, Liu Yang

**Affiliations:** 1 School of Science, Jiangsu Provincial Research Center of Light Industrial Optoelectronic Engineering and Technology, Jiangnan University, Wuxi 214122, China; lchy_1994@163.com (C.L.); huyuanda1112@jiangnan.edu.cn (Z.-D.H.); yliu_1994@163.com (L.Y.); 2 Science and Technology on Electronic Test and Measurement Laboratory, North University of China, No. 3 Xueyuan Road, Taiyuan 030051, China; shubin_yan@nuc.edu.cn; 3 School of Physics and Optoelectronic Engineering, Nanjing University of Information Science & Technology, Nanjing 210044, China; jsnanophotonics@yahoo.com

**Keywords:** plasmon-induced transparency (PIT), graphene nanoribbons (GNRs), multiple peak, graphene plasmonics

## Abstract

We present plasmonic devices, consisting of periodic arrays of graphene nanoribbons (GNRs) and a graphene sheet waveguide, to achieve controllable plasmon-induced transparency (PIT) by numerical simulation. We analyze the bright and dark elements of the GNRs and graphene-sheet waveguide structure. Results show that applying the gate voltage can electrically tune the PIT spectrum. Adjusting the coupling distance and widths of GNRs directly results in a shift of transmission dips. In addition, increased angle of incidence causes the transmission to split into multiple PIT peaks. We also demonstrate that PIT devices based on graphene plasmonics may have promising applications as plasmonic sensors in nanophotonics.

## 1. Introduction

The electromagnetically induced transparency (EIT) effect observed in atomic systems produces a sharp and transparent window in a wide range of absorption spectra range. It results from a considerable hindrance between coherent optical transitions [1,2,3,4]. In the transparency window, there is obvious normal dispersion and ultrahigh quality factor, due to which, many feasible applications in optical storage, slow light, and other nonlinear processes based on the atomic EIT have been proposed [5]. In many optical structures, analogs of EIT have been presented [6,7,8,9,10,11]. In plasmonic systems, this effect is named plasmon-induced transparency (PIT); it can be realized by the considerable hindrance between bright and dark modes [8].

Graphene is a monolayer of carbon atoms with a honeycomb-like lattice that is gaining considerable attention because of its excellent electrical and photonic properties, such as strong optical nonlinearity [12], ultrahigh electron mobility [13], and high-thermal conductivity [14]. In particular, the surface conductivity of graphene can be changed by gate voltage, electromagnetic fields, and chemical doping [15,16,17,18]. In this way, the development of tunable plasmonic devices could be promoted. Considering these characteristic properties, graphene can also support propagation of surface plasmon polaritons (SPPs). Graphene surface plasmon polaritons (GSPPs) can be excited for a broad range of frequencies from near-infrared to the terahertz region [19]. Meanwhile, due to its high-carrier mobility, GSPPs display lower propagation loss at room temperature [20]. Moreover, adjusting the chemical potential of graphene can easily influence the propagation of GSPPs. Re-fabricating the structure is not necessary anymore. There have been many applications based on graphene, including absorbers [21], electro-optical switches [22], field-effect transistors [23], amplifiers [24], diodes [25], terahertz antennas [26], filters [27], mixers [28], plasmonic Bragg reflectors [29], and so on. A graphene-based plasmonic analog to EIT nanostructures has already been numerically illustrated by Shi et al. [30]. Consequently, the graphene becomes an advanced material in tunable PIT device design.

In this study, the coupling-induced transparency with narrow waveband can be exhibited by a properly designed system based on graphene-contained plasmonic structures by numerical simulation. Compared to previous works, we show the multiple PIT phenomena with our novel asymmetrical graphene nanoribbon structures and integrally analyze the influence factors of the PIT phenomena. First, the bright and dark elements of the GNRs and graphene sheet waveguide structure are analyzed. We observe the PIT phenomenon by presenting the structure devices composed of periodic arrays of GNRs and a graphene sheet. The shift of the transmission notch occurs because of the changes of the coupling distance and the widths of GNRs. The Fermi energy strongly influences the frequencies of plasmons in graphene. In addition, multiple PIT peaks can be obtained by changing the angle of incidence. 

## 2. Materials Model and Analysis

**Figure 1:**
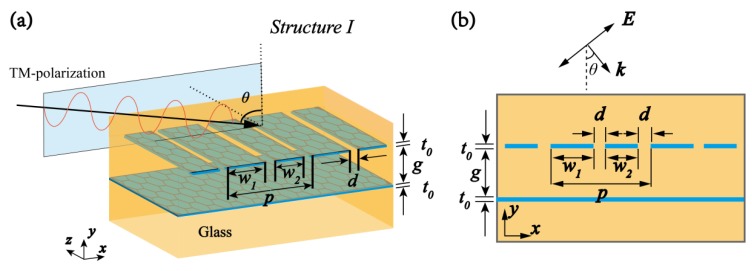
(**a**) First schematic of the proposed structure. The graphene nanoribbons (GNRs) are lying periodically above an infinite-width graphene sheet and *p* represents the cycle of the unit element. The distance between GNRs and the graphene sheet is *g*. The widths of the two adjacent GNRs are defined as *w*_1_ and *w*_2_, respectively; (**b**) Cross-sectional diagram of Structure I.

Figure 1 shows the first schematic of the structure employed in our design. The gap *g* is sandwiched by the GNRs and graphene sheet layers. (For simplicity, the surrounding material is treated as air, *ε_r_* = 1.) A transverse-magnetic (TM) polarization (magnetic field is parallel to the *y*-axis) monochromatic plane wave comes normally from the upper surface of the structure. *p* is the cycle of the unit element; *w*_1_ and *w*_2_ are the widths of the adjacent GNRs, and *d* is the distance. The designed device is easy to fabricate. The chemical vapour deposition (CVD) method can be utilized to grow multiple layers of graphene on the silica layer and to manufacture the silicon-based graphene structure waveguide. The momentum relaxation time *τ*, temperature *T*, incident wavelength *λ*, and Fermi energy *E_f_* determine the surface conductivity of graphene *σ_g_*. It follows the equation

(1) $$\sigma_{g}=\frac{ie^{2}E_{F}}{\pi \hbar^{2}(\omega +i\tau^{-1})}+\frac{ie^{2}}{4\pi \hbar }\ln\left[\frac{2\left|E_{F}\right|-(\omega +i\tau^{-1})\hbar }{2\left|E_{F}\right|+(\omega +i\tau^{-1})\hbar }\right]+\frac{ie^{2}k_{B}T}{\pi \hbar^{2}(\omega +i\tau^{-1})}2\ln\left[\exp\left(-\frac{E_{F}}{k_{B}T}\right)+1\right]$$

In our simulation, the incident light we employed is at the range of the mid-infrared region. The intraband transition contribution predominates in monolayer graphene [31]. With this understanding, the optical conductivity is given by [32]

(2) $$\sigma_{g}(\omega )=\frac{ie^{2}E_{F}/\pi \hbar^{2}}{\left(\omega +i\tau^{-1}\right)}$$

Here, *e* is the electron charge and the electron relaxation time is *τ* = *μE_F_* = *ev_F_*^2^, in which *v_F_* = *c*/300 is the Fermi velocity; *E_F_* is the Fermi energy, and *μ* = 10 m^2^/(V·S) is the direct current mobility of graphene [33,34,35]. The graphene layers are viewed as an ultrathin film with the permittivity expressed as

(3) $$\varepsilon_{g}=\varepsilon_{r}+\frac{\sigma_{g}}{\omega \varepsilon_{0}t_{0}}$$

where *t*_0_ = 1 nm is the thickness of the graphene layers. The SPPs modes, ranging from THz to the visible frequencies, could be spread over the graphene layer. The dispersion relation of the SPP modes at subwavelength scale is given by [36]

(4) $$k_{g}=k_{0}\frac{\pi \hbar^{2}\omega^{2}\varepsilon_{0}(\varepsilon_{1}+\varepsilon_{2})(1+i\pi^{-1}\tau^{-1})}{e^{2}E_{f}}$$

where *k_g_* is the in-plane wave vector in graphene, *k*_0_ is the wave vector in vacuum. *ε*_1_ and *ε*_2_ are the dielectric constants of the top and bottom surface materials of the graphene sheet, respectively. The graphene Fermi level can be tuned by adjusting the gate voltage, which can be calculated according to Equation (2).

## 3. Simulation and Results

**Figure 2:**
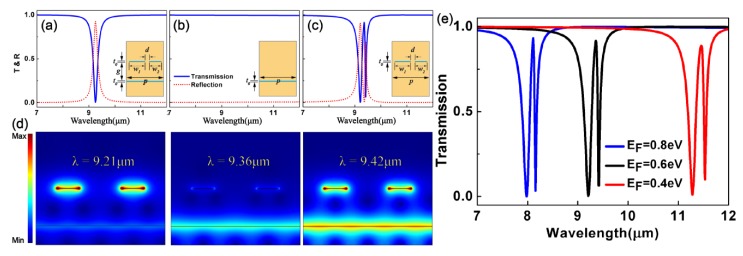
(**a**–**c**) Transmission and reflection spectra for the basic unit at normal incidence. The inset graphs are the diagrammatic sketch of graphene nanostructures under research. There are two graphene nanoribbons acting as the bright element. The graphene sheet layers are acting as the dark element. The basic unit of the graphene plasmonic structure aims to achieve plasmon-induced transparency (PIT). It is constructed by combining the bright element with the dark element. Blue areas represent graphene; (**d**) The |E| distributions corresponding to three different wavelengths; (**e**) Transmission spectra of the system for chemical potential *E_F_* = 0.4, 0.6, and 0.8 eV.

To demonstrate the graphene-based PIT phenomenon, we start with nanostructures with different radiation performances, as is shown in Figure 2a,b: bright and dark elements. The resonant plasmonic modes in the bright and dark elements separately exhibit different coupling strengths to free space. The bright modes are excited by the bright element, which consists of two GNRs lying periodically along the x direction with different: widths *w*_1_ and *w*_2_. The dark modes are simply graphene sheet layers. The separation between the GNRs and graphene sheet layers is *g*. To realize the PIT phenomenon, the basic unit of the structure can be formed by combining the bright element with the dark element.

To study the PIT phenomenon in graphene nanostructures, we set up a graphene PIT structure, which is constructed by combining the coupled bright element with the dark element as the basic unit. The units array periodically with a period *p* = 1200 nm. Figure 2c shows the transmission and reflection spectra that we simulated at normal incidence for the graphene PIT structure.

Obviously, a PIT-like transparent window can be observed from the transmission spectrum of the graphene PIT structure. The two notches’ wavelengths are at 9.21 and 9.42 μm, and the peak is positioned at 9.36 μm. This phenomenon is essentially analogous to the EIT spectrum in atomic systems. At the PIT peak, almost no reflection can be observed. At the right transmission dip, the reflection is obviously low, revealing a strong absorption. In addition, to make the disturbance between the dark and bright modes visible, we exhibit the |E| distributions at these concerned wavelengths. The dipole mode can be effectively excited at the transparent peak by the incident light of 9.36 μm, while the excitation of the quadruple mode is prevented in the dark element. However, the coupling between the bright and dark modes can indirectly inspire the dark mode. The dark mode will then couple to the bright mode again. It is noticed that a phase difference of *π* occurs between the channels of the direct and indirect excitations [8,37]. Therefore, there is a mutual destructive interference between the bright and dark modes. Furthermore, the fields in the bright element are restrained, which causes a transparent window, similar to the EIT phenomenon in the atom. By comparison, the interference is beneficial to the enhancement of the fields in both elements at the transmission notches.

The optical performances of graphene can be considerably improved by doping or gating as plasmons in graphene are greatly dependent on the Fermi energy. By adjusting the Fermi energy, the electron density in graphene can be changed, resulting in a change in the resonance frequencies of graphene plasmonic nanostructures. As a result, the PIT spectrum of the graphene PIT structure can be dynamically controlled over a wide range of wavelengths. Such tunability can be hard to realize in atomic EIT or other PIT systems. The transmission spectra for structure I at different *E_F_* are plotted in Figure 2e. The PIT transparency windows can be blue-shifted obviously while *E_F_* changes from 0.4 to 0.8 eV. Therefore, the PIT spectrum can be shifted over a rather wide range of wavelengths by tuning the Fermi energy.

**Figure 3:**
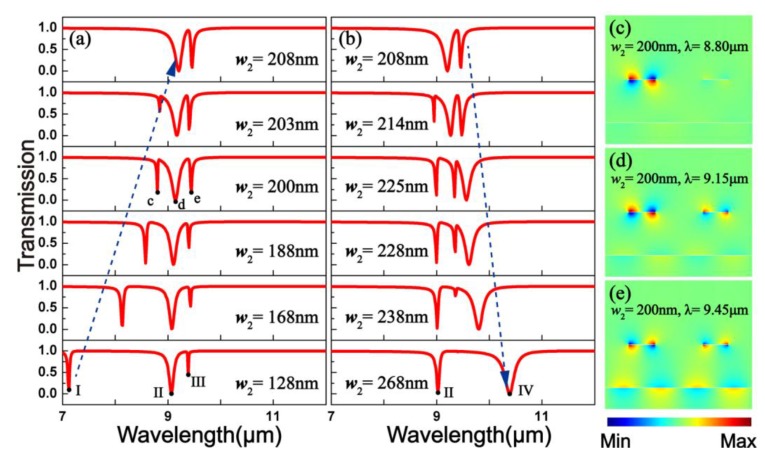
(**a**,**b**) Computerized ionospheric tomography transmission spectra at different widths *w*_2_. Here, *p* = 1200 nm, *w*_1_ = 208 nm, *g* = 350 nm, and *E_F_* = 0.6 eV; (**c**–**e**) Field distributions *E_y_* of the transmission dips.

The transmission spectra of structure I are shown in Figure 3a,b. Here, *p* = 1200 nm, *w*_1_ = 208 nm, *g* = 350 nm, and *E_F_* = 0.6 eV. As the width *w*_2_ is increased, dip I keeps red-shifting, whereas the position of dip II remains almost invariant. When *w*_2_ = 200 nm, double PIT peaks can be achieved. Dip I is integrated into dip II as *w*_2_ increased from 200 to 208 nm. Only one PIT peak can then be observed. When *w*_2_ > 208 nm, dip IV is continuously red-shifting and ultimately the only PIT peak disappears. Furthermore, Figure 3c–e show the field distributions of *E_y_* consistent with the transmission dips under *w*_2_ = 200 nm. The super resonance modes (symmetric and antisymmetric modes) can be found at the transmission dips by the incident light of *λ* = 8.80, 9.15, and 9.45 μm. At that resonance wavelength, the simultaneous excitement of the two super resonances leads to near-field curtailment in the bright element and enhancement in the dark mode. The two resonant structures are extremely excited and the PIT can be realized when those two resonance modes are at the same wavelength.

**Figure 4:**
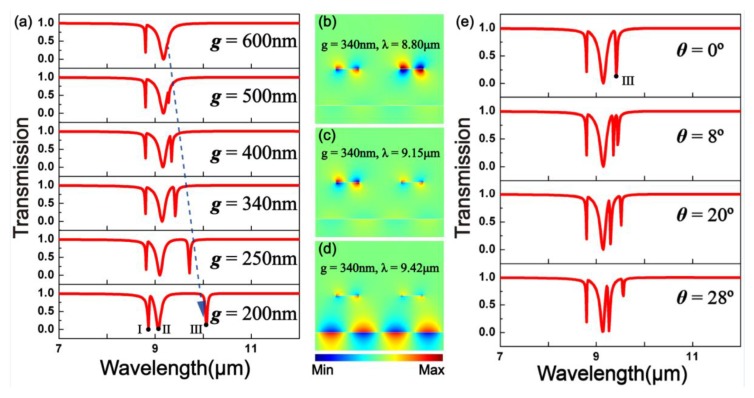
(**a**) Transmission spectra of structure I at different gap *g* values between the graphene sheet and the GNRs; (**b**–**d**) The field distributions *E_y_* of the transmission dips under *g* = 340 nm; (**e**) Spectral responses of structure I with different angles of incidence.

The transmission spectra of structure I at different gap *g* values between the graphene sheet and the GNRs are plotted in Figure 4a. As the gap *g* shrinks, dip II splits into two notches, and dip III keeps red-shifting while *g* < 600 nm. Figure 4b–d show the field distributions of *E_y_* corresponding to the transmission dips under *g* = 340 nm. The super resonance modes (symmetric and antisymmetric modes) at *λ* = 8.80, 9.15, and 9.45 μm related to the transmission dips can also be found. Figure 4e shows the spectral responses of structure I that are greatly influenced by the angle of incidence while *g* = 340 nm keeps invariable. With the angle of incidence increasing, dip III splits into two notches, with one blue-shifted and the other red-shifted and triple PIT peaks can be achieved.

**Figure 5:**
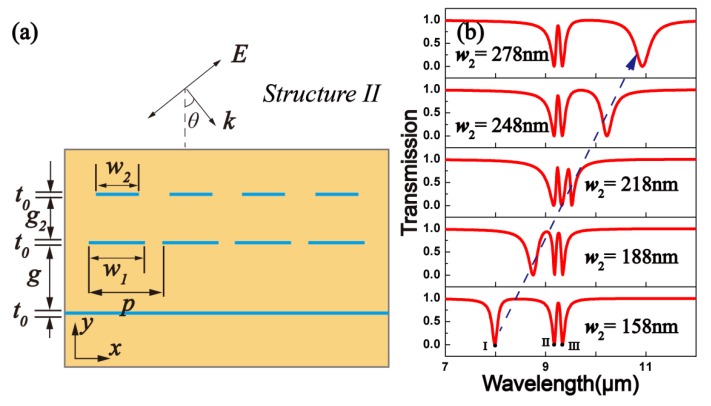
(**a**) Cross-sectional diagram of the second structure proposed; (**b**) Computerized ionospheric tomography transmission spectra at different widths *w*_2_. Here, *p* = 600 nm, *w*_1_ = 208 nm, *g* = 350 nm, and *E_F_* = 0.6 eV.

Furthermore, the second schematic of the structure employed in our design is shown in Figure 5a. The distance between the first layer of GNRs and graphene sheet layers is *g*. The first and the second layers of GNRs are separated by the gap *g*_2_. In one period, the two graphene nanoribbons are on the same symmetry axis. Their widths are defined as *w*_1_ and *w*_2_, respectively. A TM polarization (magnetic field is parallel to the *y*-axis) monochromatic plane wave comes normally from the upper surface of structure II. The transmission spectra of the structure are shown in Figure 5b. Here, *p* = 600 nm, *w*_1_ = 208 nm, and *E_F_* = 0.6 eV. As the width *w*_2_ increased, we can see a gradual red-shift of dip I. Especially, while *w*_2_ = 218 nm, double PIT peaks can be obtained.

**Figure 6:**
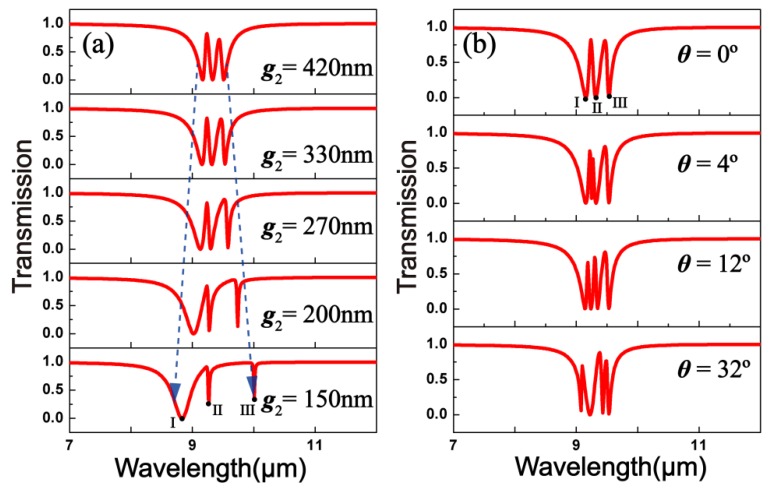
(**a**) Transmission spectra of structure II at diverse gap *g*_2_ values between the first and the second layers of GNRs; (**b**) Spectral responses of structure II at different angles of incidence.

In addition, the transmission spectra of structure II that we calculated at different gap *g*_2_ values are plotted in Figure 6a. As can be clearly observed, dip I is blue-shifting, whereas dip II is red-shifting as *g*_2_ shrinks. When *g*_2_ = 330 nm, double PIT peaks can be observed. While *g* = 330 nm keeps invariable, the angular-dependent transmission spectra of structure II are plotted in Figure 6b. With the angle of incidence increasing, dip II splits into two notches, with one blue-shifted and the other red-shifted and triple PIT peaks can be achieved.

## 4. Conclusions

We studied the radiative properties of graphene nanostructures by numerical simulations. Periodic graphene strips can serve as the bright element; a graphene sheet parallel to the periodic graphene strips can serve as the dark element. A graphene PIT structure can be constructed by combining the bright element with the dark element; it shows a PIT spectrum similar to the atomic EIT. Significantly, changing the gate voltage can directly tune the PIT spectrum. The position of the transmission notch greatly depends on the changes of the coupling distance and the widths of GNRs. With the appropriate distance and widths, double PIT peaks can be obtained. In addition, multiple PIT peaks can be achieved by changing the angle of incidence. Our study indicates that the demonstrated PIT devices based on graphene plasmonics may pave a new way for the development of plasmonic sensors in nanophotonics applications.

## References

[1] Harris S.E. (1997). Electromagnetically induced transparency. Phys. Today.

[2] Gu J.Q., Singh R., Liu X., Zhang X., Ma Y., Zhang S., Maier S.A., Tian Z., Azad A.K., Chen H.-T. (2012). Active control of electromagnetically induced transparency analogue in terahertz metamaterials. Nat. Commun..

[3] Tassin P., Zhang L., Zhao R., Jain A., Koschny T., Soukoulis C.M. (2012). Electromagnetically induced transparency and absorption in metamaterials: The radiating two-oscillator model and its experimental confirmation. Phys. Rev. Lett..

[4] Boller K.J., Imamoğlu A., Harris S.E. (1991). Observation of electromagnetically induced transparency. Phys. Rev. Lett..

[5] Fleischhauer M., Imamoglu A., Marangos J.P. (2005). Electromagnetically induced transparency: Optics in coherent media. Rev. Mod. Phys..

[6] Xu Q.F., Sandhu S., Povinelli M.L., Shakya J., Fan S., Lipson M. (2006). Experimental realization of an on-chip all-optical analogue to electromagnetically induced transparency. Phys. Rev. Lett..

[7] Papasimakis N., Fedotov V.A., Zheludev N.I., Prosvirnin S.L. (2008). Metamaterial analog of electromagnetically induced transparency. Phys. Rev. Lett..

[8] Zhang S., Genov D.A., Wang Y., Liu M., Zhang X. (2008). Plasmon-induced transparency in metamaterials. Phys. Rev. Lett..

[9] Yang X.D., Yu M., Kwong D.-L., Wong C.W. (2009). All-optical analog to electromagnetically induced transparency in multiple coupled photonic crystal cavities. Phys. Rev. Lett..

[10] Tassin P., Zhang L., Koschny T., Economou E.N., Soukoulis C.M. (2009). Low-loss metamaterials based on classical electromagnetically induced transparency. Phys. Rev. Lett..

[11] Sun Y., Jiang H., Yang Y., Zhang Y., Chen H., Zhu S. (2011). Electromagnetically induced transparency in metamaterials: Influence of intrinsic loss and dynamic evolution. Phys. Rev. B..

[12] Wright A.R., Xu X.G., Cao J.C., Zhang C. (2009). Strong nonlinear optical response of graphene in the terahertz regime. Appl. Phys. Lett..

[13] Bolotin K.I., Sikes K.J., Jiang Z., Klima M., Fudenberg G., Hone J., Kim P., Stormer H.L. (2008). Ultrahigh electron mobility in suspended graphene. Solid State Commun..

[14] Balandin A.A., Ghosh S., Bao W., Calizo I., Teweldebrhan D., Miao F., Lau C.N. (2008). Superior thermal conductivity of single-layer graphene. Nano Lett..

[15] Bao Q.L., Loh K.P. (2012). Graphene photonics, plasmonics, and broadband optoelectronic devices. ACS Nano.

[16] Grigorenko A.N., Polini M., Novoselov K.S. (2012). Graphene plasmonics. Nat. Photonics.

[17] Wang X.S., Chen C., Pan L., Wang J. (2016). A graphene-based Fabry-Pérot spectrometer in mid-infrared region. Sci. Rep. UK.

[18] Song C., Xia X., Hu Z., Liang Y., Wang J. (2016). Characteristics of plasmonic Bragg reflectors with graphene-based silicon grating. Nanoscale Res. Lett..

[19] Li Z.B., Yao K., Xia F., Shen S., Tian J., Liu Y. (2015). Graphene plasmonic metasurfaces to steer infrared light. Sci. Rep. UK.

[20] Brar V.W., Jang M.S., Sherrott M., Lope J.J., Atwater H.A. (2013). Highly confined tunable mid-infrared plasmonics in graphene nanoresonators. Nano Lett..

[21] Yao G., Ling F., Yue J., Luo C., Luo Q., Yao J. (2016). Dynamically electrically tunable broadband absorber based on graphene analog of electromagnetically induced transparency. IEEE Photonics J..

[22] Li Y., Yu H., Dai T., Jiang J., Wang G., Yang L., Wang W., Yang J., Jiang X. (2016). Graphene-based floating-gate nonvolatile optical switch. IEEE Photonics Technol. Lett..

[23] Fahad M.S., Srivastava A., Sharma A.K., Mayberry C. (2016). Analytical current transport modeling of graphene nanoribbon tunnel field-effect transistors for digital circuit design. IEEE Trans. Nanotechnol..

[24] Yurchenko S.O., Komarov K.A., Pustovoit V.I. (2015). Multilayer-graphene-based amplifier of surface acoustic waves. AIP Adv..

[25] Al-Dirini F., Mohammed M.A., Hossain F.M., Nirmalathas T., Skafidas E. (2016). All-graphene planar double-quantum-dot resonant tunneling diodes. IEEE J. Electron Devices Soc..

[26] Wang X.C., Zhao W.S., Hu J., Yin W.Y. (2015). Reconfigurable terahertz leaky-wave antenna using graphene-based high-impedance surface. IEEE T. Nanotechnol..

[27] Correas-Serrano D., Gomez-Diaz J.S., Perruisseau-Carrier J., Alvarez-Melcon A. (2014). Graphene-based plasmonic tunable low-pass filters in the terahertz band. IEEE Trans. Nanotechnol..

[28] Mao X., Cheng C., Huang B., Zhang Z., Gan S., Chen H., Chen H. (2015). Optoelectronic mixer based on graphene FET. IEEE Electr. Device Soc..

[29] Wang Y., Chen Q., Shen X. (2015). Actively controlled plasmonic Bragg reflector based on a graphene parallel-plate waveguide. AIP Adv..

[30] Shi X., Han D., Dai Y., Yu Z., Sun Y., Chen H., Liu X., Zi J. (2013). Plasmonic analog of electromagnetically induced transparency in nanostructure graphene. Opt. Express.

[31] Hanson G.W. (2008). Quasi-transverse electromagnetic modes supported by a graphene parallel-plate waveguide. J. Appl. Phys..

[32] Li H.J., Wang L.L., Sun B., Huang Z.R., Zhai X. (2014). Controlling mid-infrared surface plasmon polaritons in the parallel graphene pair. Appl. Phys. Express.

[33] Wang L., Li W., Jiang X.Y. (2015). Tunable control of electromagnetically induced transparency analogue in a compact graphene-based waveguide. Opt. Lett..

[34] Lin Q., Zhai X., Wang L., Wang B., Liu G., Xia S. (2015). Combined theoretical analysis for plasmon-induced transparency in integrated graphene waveguides with direct and indirect couplings. EPL Europhys. Lett..

[35] Wang B., Zhang X., Yuan X., Teng J. (2012). Optical coupling of surface plasmons between graphene sheets. Appl. Phys. Lett..

[36] Jablan M., Buljan H., Soljacic M. (2009). Plasmonics in graphene at infrared frequencies. Phys. Rev. B.

[37] Lu Y., Xu H., Rhee J.Y., Jang W.H., Ham B.S., Lee Y. (2010). Magnetic plasmon resonance: Underlying route to plasmonic electromagnetically induced transparency in metamaterials. Phys. Rev. B.

